# *Bordetella pertussis* in infants hospitalized for acute respiratory symptoms remains a concern

**DOI:** 10.1186/1471-2334-13-526

**Published:** 2013-11-08

**Authors:** Ambra Nicolai, Raffaella Nenna, Paola Stefanelli, Anna Carannante, Concetta Schiavariello, Alessandra Pierangeli, Carolina Scagnolari, Corrado Moretti, Paola Papoff, Enea Bonci, Marianna Ferrara, Stefano Papasso, Fabio Midulla

**Affiliations:** 1 Department of Paediatrics, “Sapienza” University of Rome, Rome, Italy; 2 Department of Infectious, Parasitic & Immune-mediated Diseases, Istituto Superiore di Sanità, Rome, Italy; 3 Molecular Medicine Department, “Sapienza” University of Rome, Rome, Italy

**Keywords:** Bronchiolitis, *Bordetella pertussis*, Infants, Vaccine

## Abstract

**Background:**

Preliminary results suggest that pertussis infection might be considered in infants during a seasonal respiratory syncytial virus (RSV) outbreak.

**Methods:**

In order to analyze clinical features and laboratory findings in infants with pertussis hospitalized for acute respiratory symptoms during a seasonal RSV outbreak, we conducted a retrospective single-center study on 19 infants with pertussis (6 boys; median age 72 days) and 19 matched controls (RSV-bronchiolitis), hospitalized from October 2008 to April 2010. *B. pertussis* and RSV were detected from nasopharyngeal washes with Real Time-PCR.

**Results:**

Infants with pertussis were less often breastfeed than infants with RSV bronchiolitis (63.2% vs 89.5%; p <0.06). Clinically, significantly fewer infants with pertussis than controls had more episodes of whooping cough (63.2% vs 0.0%; p < 0.001) and also less frequently fever at admission (15.8% vs 68.4%; p <0.01), apnea (52.6% vs 10.5%; p <0.006), and cyanosis (52.6% vs 10.5%; p < 0.006). Infants with pertussis had more often no abnormal chest sounds on auscultation than infants with RSV bronchiolitis (0% vs 42,1%; p < 0.005). The absolute blood lymphocyte and eosinophil counts were higher in infants with *B. pertussis* than in controls with bronchiolitis (23886 ± 16945 vs 10725 ± 4126 cells/mm^3^, p < 0.0001 and 13.653 ± 10.430 vs 4.730 ± 2.400 cells/mm^3^, p < 0.001). The molecular analysis of 2 *B. pertussis* isolates for *ptxA1*, *ptxP3*, and *prn2* genes showed the presence of gene variants.

**Conclusions:**

When infants are hospitalized for acute respiratory symptoms, physicians should suspect a pertussis infection, seek for specific clinical symptoms, investigate lymphocyte and eosinophil counts and thus diagnose infection early enough to allow treatment.

## Background

Pertussis is a major public health problem, affecting adolescents and adults as well as children. Despite a widespread vaccination program, over the past fifteen years was seen a return of pertussis worldwide [1]. Pertussis resurgence in Europe has been attributed to an incomplete immunization program or to genetic changes in *Bordetella pertussis* (*B. pertussis*). [2] The currently used acellular pertussis vaccine contains the pertactin gene variant *prn1* and the pertussis toxin-B S1 subunit [3,4]. Molecular changes in these two genes over the past years suggest that the antigenic divergence may make pertussis vaccination less effective than before [5,6].

In some countries pertussis immunization is not mandatory and in others vaccination schedules suggest the first dose to be given at the age of 3 months. Hence, some infants remain unimmunized or incompletely immunized. In those who are incompletely immunized pertussis may develop in an atypical clinical form and be difficult to diagnose. Pertussis can be especially difficult to diagnose in children under 1 year of age during winter season, when other pathogens, such as respiratory syncytial virus (RSV), circulate. In these difficult cases, pertussis acute respiratory symptoms can overlap with those of bronchiolitis. A study conducted in a group of infants hospitalized for RSV bronchiolitis showed that almost 2% of the patients were co-infected with *B. pertussis*[7,8]. Since *B. pertussis*-RSV co-infection is infrequent in young infants, physicians should keep the possibility of co-infections in mind as to diagnose it early and prevent bronchiolitis from becoming more severe [9-12].

Although the standard diagnostic criterion for identifying *B. pertussis* is culture obtained from nasal swabs or nasopharyngeal aspirates, confirmatory information comes nowdays from molecular techniques such as real time-polymerase chain reaction (RT-PCR). Usually, in clinical practice the diagnosis is generally reached without microbiological confirmation [13,14]. What we conspicuously lack is the clinician’s awareness of the clinical and laboratory data needed to reach a suspected *B. pertussis* diagnosis in order to start treatment early.

The main purposes in our retrospective, single-center study were to describe and compare clinical and laboratory features in infants with pertussis infection to infants hospitalized for RSV bronchiolitis, and to analyze the genetic characteristics of *B. pertussis*.

## Methods

### Patients

In a retrospective single-center study, from a group of infants hospitalized from October 2008 to April 2010 at our Pediatric Emergency Department for acute respiratory symptoms we selected for study 19 consecutive infants aged less than 12 months (6 boys, median age 72 days, range 20-187) with Real Time-PCR confirmed pertussis. We also analyzed data for *B. pertussis* variants among hospitalized patients in whom *B. pertussis* was cultured. As a control group, we recruited 19 age- and sex-matched infants (6 boys, median age 71 days, range 20-183) from 164 infants, hospitalized during the same period with RT-PCR confirmed RSV bronchiolitis and negative for *B. pertussis*. The diagnosis of bronchiolitis was considered in infants less than 12 months with the first episode of acute infection of the lower respiratory tract. Infants with co-morbidity were excluded.

Detailed demographic, clinical and laboratory data were obtained from patients’ parents with a structured questionnaire and from medical files. Clinical outcome variables evaluated included gender, gestational age, birth weight, type of delivery, DTaP (diphtheria, tetanus and acellular pertussis) vaccination received, breast feeding history, age and weight at admission, number of siblings, siblings’ schooling, cough at admission (presence and duration), paroxysmal cough, presence of fever (body temperature >37.5°C), apnea and cyanosis, chest sounds, and hospitalization days. Laboratory outcome variables investigated were white blood-cell count (WBC), lymphocyte count, eosinophil count, neutrophil count, platelet count, hemoglobin (Hb), glutamic oxaloacetic transaminase (SGOT), glutamic pyruvic transaminase (SGPT), gamma-glutamyl transferase (GGT) and C-reactive protein (CRP). At hospital admission, each infant was assigned a clinical severity score ranging from 0 to 8, according to respiratory rate (<45/min = 0, 45-60/min = 1, >60/min = 2), arterial oxygen saturation in room air (> 95% = 0, 95-90% = 1, < 90% = 2), retractions (none = 0, present = 1, present + nasal flare = 2), and ability to feed (normal = 0, reduced = 1, intravenous fluid replacement =2) [15].

All infants’ parents were asked to participate in the study and gave written informed consent. The study was approved by the Policlinico Umberto I institutional review board (Reference n° 2377/09.02.2012).

### Nasal pharyngeal washing

From 1 to 3 days after hospitalization, all infants underwent nasal pharyngeal washing with 3 ml of sterile saline injected in each nostril and collected with a syringe. All samples were delivered at room temperature within 1-2 hours to the Department of Infectious, Parasitic & Immune-mediated Diseases at the Istituto Superiore di Sanità Rome to identify *B. pertussis* and to the Molecular Medicine Department, “Sapienza” University of Rome (Virology Laboratory), to identify RSV.

### *B. pertussis* detection

Pertussis was diagnosed using the Insertion Sequence (IS) IS481, and the *ptxP* gene as “target” genes [16]. For RealTime-PCR the SYBR Green Detection assay was performed using the LightCycler 2.0 (Roche Diagnostic). Data were analyzed with LightCycler software (version 4.0, Roche Diagnostic). All samples testing positive for *B. pertussis* were confirmed with the “*Bordetella* Real Time PCR” kit (Diagenode, Belgium). The specific primers and probes for detecting *B. pertussis* DNA with the commercially-available PCR kit (Diagenode, Belgium) were selected from the literature [17].

*B. pertussis* isolates were cultured on charcoal agar plates (Oxoid England) containing defibrinated sheep blood at 10% and incubated at 35°C up to 7 days, and inspected daily. The isolates were stored at -80°C in medium containing brain heart infusion (BHI, Oxoid England) supplemented with 15% glycerol. The chromosomal DNA from each *B. pertussis* strain was extracted using the QIAamp DNA minikit (QiaGEN, Hilden, Germany). To obtain gene molecular sequence data, the pertussis toxin S1 subunit (*ptxA*), pertussis toxin promoter (*ptxP*) and pertactin (*prn*) sequences were analyzed for two *B. pertussis* clinical isolates. The used primers are those described previously [2,18,19].

All the amplifications were done with the Mastercycler personal thermal cycler (Eppendorff, Hamburg, Germany). PtxA, ptxP and prn were amplified in 50 μl containing 5 μl of PCR buffer 10X (containing 15 mM of MgCl_2_), 50 mM MgSO_4_, 0.01 mM deoxynucleotide (dNTP, Finnzymes, Finlandia), 50 pmol of each primer and 0.5 U of HotStarTaq (QIAGEN, Hilden, Germania); 10% dimethyl sulfoxide was added also for the prn gene. The ptxA and ptxP genes were amplified with 35 cycles at 94°C for 30 s; 64°C (ptxA) and 60°C (ptxP) for 30 s and 72°C for 30 s; and the prn gene with 30 cycles at 95°C for 20 s, 55°C for 30 s and 72°C for 1 min. All amplification products were analyzed by electrophoresis and purified with “QIAquick purification columns” kit (QIAGEN) for subsequent sequence analysis. Sequences were analyzed with BLAST program. The amino acid sequences resulting from the nucleotide sequences were compared using the DNAMAN program (version 5.2.10).

### RSV detection

Upon arrival in the virological laboratory, the nasal washings were centrifuged to remove the mucus present in the sample and then divided into two aliquots. The first aliquot was used for nucleic acid extraction using a total nucleic acid isolation kit (Roche Diagnostics, Mannheim, Germany) and an RT-PCR panel that sought RSV and 13 more respiratory viruses: influenza A and B viruses, human coronavirus 0C43, 229E, NL-63, HUK1, adenovirus, parainfluenza virus 1-3, human-metapneumovirus, human-Bocavirus, and rhinovirus [14].

### Statistical analysis

All data were analyzed using the SPSS program (version 17.0). Data were reported as median, range, mean, standard deviation, and percentage. Categorical variables were analyzed with Pearson chi-square (χ^2^) test and Fisher test continuous variables with the Mann-Whitney U test. P values ≤ 0.05 were considered to indicate statistical significance.

## Results

No *B. pertussis* clustering was observed during the study. No significant differences were found between infants with confirmed *B. pertussis* and control infants with RSV-bronchiolitis for demographic characteristics including gender, gestational age, birth weight, type of delivery, DTaP vaccination status, age at admission, weight on admission, presence of siblings and schooling. The percentage of infants who were breast fed at hospitalization was lower in the group of infants with *B. pertussis* than in the one with RSV bronchiolitis (63.2% vs 89.5%, p <0.06) (Table 1).

**Table 1:** Demographic characteristics of the 19 infants with pertussis and the 19 infants with RSV bronchiolitis

**Demographic characteristics**	**Infants with pertussis**	**Infants with RSV bronchiolitis**	**p**
Age at admission (days)	71.5 ± 48.0	71.4 ± 48.0	n.s.
Male:Female	6:13	6:13	n.s.
Siblings n° (%)	14 (73.7%)	12 (63.2%)	n.s.
Schooling n° (%)	11 (57.9%)	12 (63.2%)	n.s.
Gestational age median in weeks (range)	40 (36-42)	39 (37-42)	n.s.
Spontaneous delivery	12 (63.2%)	9 (57.9%)	n.s.
Weight at birth median in kg (range)	3.220 (2.100-4.000)	3.300 (2.130- 3.850)	n.s.
Weight at admission median in kg (range)	4.290 (2.820-7.850)	4.900 (3.230-7.730)	n.s.
Breast feeding at admission n° (%)	12 (63.2%)	17 (89.5%)	0.06
Breast feeding median in days (range)	20 (0-150)	30 (0-94)	n.s.
Vaccination (DTaP)			n.s.
1^st^ dose	4 (21.0%)	4 (21.1%)	
2^nd^ dose	1 (5.3%)	0	

p values by Mann-Whitney U test.

*RSV*: respiratory syncytial virus.

Data for the clinical characteristics showed that the percentage of infants with paroxysmal cough was significantly higher and cough at admission lasted longer in infants with pertussis than in control infants (63.2% vs 0.0%, p <0.001, 7 days vs 3 days, p <0.09). The percentage of infants with fever (TC > 37.5°C) was significantly lower in infants with pertussis than in the control group (15.8% vs 68.4%, p <0.001). The percentage of infants with apnea and cyanosis was significantly higher in infants with pertussis than in those with RSV bronchiolitis (apnea 52.6% vs 10.5%, p <0.006 and cyanosis 52.6% vs 10.5%, p <0.006). Finally, infants with pertussis less often had abnormal chest sounds on auscultation than infants with RSV bronchiolitis (0% vs 42,1%; p < 0.005). No significant difference was found between the two groups regarding clinical severity scores [median(range) 3(0-6) vs 5(0-7)] and days hospitalization [7(2-15) vs 5(1-27)]. Clinical complications rarely arose in the two groups: two infants in the pertussis group (10.5%) and only one infant in the bronchiolitis group (5.3%) had bradycardia (p = n.s.). Respiratory complications (pneumothorax) arose in three of the 19 patients with pertussis (15.8%) and in only one of the 19 with bronchiolitis (5.3%) (p = n.s.) (Table 2). No significant differences were found between the two groups for chest radiograph findings (air trapping, peribronchial thickening and consolidations).

**Table 2:** Clinical characteristics of the 19 infants with pertussis and the 19 infants with RSV bronchiolitis

**Clinical characteristics**	**Infants with pertussis**	**Infants with RSV bronchiolitis**	**p**
Paroxysmal cough	12 (63.2%)	0 (0.0%)	0.001
Cough at admission median in days (range)	7 (0-20)	3 (0-15)	0.09
Apnea	10 (52.6%)	2 (10.5%)	0.006
Cyanosis	10 (52.6%)	2 (10.5%)	0.006
Fever (> 37.5°C)	3 (15.8%)	13 (68.4%)	0.001
Chest sounds			0.005
Rales	7 (36.8%)	11 (57.9%)	
Wheeze	3 (15.8%)	2 (10.5%)	
Rales and Wheeze	1 (5.3%)	6 (31.6%)	
Negative	8 (42.1%)	0 (0.0%)	
Severity score median (range)	3 (0-6)	5 (0-7)	n.s.
Hospitalization length median in days (range)	7 (2-15)	5 (1-27)	n.s.
Complications			n.s.
Bradycardia	2 (10.5%)	1 (5.3%)	
Respiratory	3 (15.8%)	1 (5.3%)	
Total	5 (26.6%)	2 (10.5%)	

p values by Mann-Whitney U test.

*RSV*: respiratory syncytial virus**.**

Laboratory data showed significantly higher absolute WBC and lymphocyte numbers in children with pertussis than in infants with RSV bronchiolitis (23886 ± 16945, vs 10725 ± 4126 cells/mm^3^, p <0.0001 and 13.653 ± 10.430 vs 4.730 ± 2.400 cells/mm^3^, p <0.001 by Mann-Whitney U test). Finally, children with pertussis had higher absolute blood eosinophil numbers than children with RSV bronchiolitis (510 ± 500 vs 86 ± 123 cells/mm^3^ p <0.001). No significant differences were reported for absolute neutrophil numbers, absolute platelet numbers, Hb concentration, SGOT, SGPT, GGT, and CRP (Table 3).

**Table 3:** Laboratory characteristics of the 19 infants with pertussis and the 19 infants with RSV bronchiolitis

**Laboratory characteristics**	**Infants with pertussis**	**Infants with RSV bronchiolitis**	**p**
White blood cells (n/mm^3^) median (range)	18.760 (6.760-73790)	9.815 (4.770-20.910)	0.001
Lymphocyte count (n/mm^3^) median (range)	9.290 (4.320-38.665)	4.155 (1.803-8.366)	0.001
Neutrophil count (n/mm^3^) median (range)	4.500 (1.470-27.121)	4.170 (992-11.160)	n.s.
Eosinophil count (n/mm^3^) median (range)	375 (14-1.770)	45 (0-527)	0.001
Hemoglobin (mg/dl) median (range)	12.1 (10.2-15.9)	11.15 (9.40-16.60)	n.s.
Platelets (n/mm^3^) median (range)	576.000 (315.000-1.180.000)	507.000 (300.000-1.045.000)	n.s.
C-reactive protein (mg/dl) median (range)	0.13 (0.01-2.35)	0.62 (0-2.35)	n.s.
SGOT (UI/L) median (range)	39 (20-103)	41 (17-90)	n.s.
SGPT (UI/L) median (range)	31 (13-92)	23.5 (13-60)	n.s.
GGT (UI/L) median (range)	43 (13-166)	28 (7-117)	n.s.

*RSV*: respiratory syncytial virus; *SGOT*: Glutamic oxaloacetic transaminase; *SGPT*: glutamic pyruvic transaminase; *GGT*: gamma-glutamyl transferase.

p values by Mann-Whitney U test.

Of the 19 infants with pertussis, four (21.0%) had received the first DTaP vaccination dose and only one (5.3%) had received two doses. In the control group, 4 infants (21.0%) received the first DTaP vaccination dose (Table 1). Of the 19 patients who tested positive for *B. pertussis*, 13 (68.4%) had already undergone macrolide antibiotic therapy when specimens were collected, 4 (21.0%) had specimens collected after antibiotic therapy, and 2 (10.5%) received no antibiotic therapy. Three of the 19 infants with pertussis infection (15.8%) were co-infected with RSV and two (10.5%) with Rhinovirus. No differences were found for clinical and demographic findings between infants with *B. pertussis* infection alone and infants with *B. pertussis* and RSV or RV co-infection.

### Molecular analysis of pertussis cases

All 19 cases of suspected pertussis were confirmed by molecular analysis, of which for 2 *B. pertussis* strains have been isolated and analyzed for the evaluation of molecular variants. In particular, molecular analysis detected *ptxA1*, *ptxP3* and *prn2* allele variants (Table 4). Only 2 *B. Pertussis* strains were cultivated because 13 infants enrolled were already receiving specific antibiotic therapy when the specimen was collected or because technical problems arose during sample collection.

**Table 4:** **Genetic analysis for the two cultured *B. pertussis *** **isolated**

**Patient**	**Age (months)**	**Sex**	**Vaccination**	**Macrolide therapy**	***ptxA***	***ptx*-P**	***prn***
**1**	1	F	No	No	*ptxA1*	*ptx*-*P3*	*prn*-*2*
**2**	5	M	No	No	*ptxA1*	*ptx*-*P3*	*prn*-*2*

*ptxA1*: pertussis toxin subunit 1, *ptxP*: pertussis toxin promoter; *prn*: pertactin.

## Discussion

Our retrospective single-center study identified several demographic, clinical and laboratory features that might help in diagnosing suspected pertussis in infants hospitalized for acute respiratory symptoms. Another important finding in this study is that the *B. pertussis* strains circulating in our Pediatric Emergency Department seem to differ genetically from those currently used in DTaP vaccines. Of the 38 hospitalized infants enrolled in our study, five with pertussis and four with bronchiolitis had already been vaccinated. In Italy, vaccination schedule provides a first pertussis vaccine dose at the age of 3 months, and the booster doses at 5 and 12 months of age. This finding confirms previous reports, showing that many children with pertussis have never been vaccinated against pertussis, and that the first dose (or the first two doses) often provides scarce protection [14,20-25].

When we analyzed our patients’ demographic data, in line with others we found that children with whooping cough were breastfed less frequently than those with bronchiolitis [26,27]. These results prevent us from conjecturing whether breast feeding might protect against the risk of *B. pertussis* infection in the first year of life and call for other studies to assess this important question [27].

Our study once again confirms that clinical data can help in diagnosing whooping cough diagnosis in infants [12,15,28]. As expected, the hallmark clinical characteristic specific for pertussis, is paroxysmal cough. In this study we confirmed that other clinical signs suggesting *B. pertussis* infection are episodes of apnea and cyanosis [4,8,15,28]. On physical examination, fever is absent in children with pertussis, and more common in patients with bronchiolitis. In our study sample, on chest auscultation we found that over 40% of the children with pertussis had no abnormal chest sounds, whereas all the patients in control group had rales.

In the 19 infants hospitalized for suspected *B. pertussis* we studied, when clinical features suggested whooping cough as a diagnosis, additional proof might come from laboratory findings showing marked lymphocytosis [12,15,28,29]. Another distinctive finding was the significantly higher eosinophil numbers in infants with pertussis than in the control group. Since the early 1960-1970s, research has questioned the role of *B. pertussis* and its toxin in the hyper-eosinophilia response to pertussis [30-32], but the hypothesis awaits confirmatory data from other studies and research.

In two pertussis isolates, we undertook genetic molecular analysis to investigate gene variation in the pertussis toxin gene (*ptx*), in particular its S1 subunit (*ptxA*), the pertussis toxin gene promoter (*ptx-P*), and *prn*[4-6]. The “universal” indicator for characterizing the molecular features of *B. pertussis* is the catalytic subunit S1*.* Four S1 subunit variants have been described: S1-A (*ptxA1*), S1-B (*ptxA2*), S1-D (*ptxA3*), and S1-E (*ptxA4*) [2,4,6,16,18,19]. Culture and molecular data allowed us to identify strains that express the S1-A variant, whereas pertussis vaccine contains the S1-B variant [4]. Pertactin gene polymorphisms reside in regions called R1 and R2. As many as eleven different *prn* alleles have been identified [5,6]. Acellular pertussis vaccine contains the *prn*-1 allele. In the two infants whose aspirates were suitable for molecular genetic analysis, RT-PCR identified the *prn-2* gene, also found in infants from other European countries (France and Finland) [5,6] and in Japan [4]. We also found in both strains the *ptx-P3* variant of the pertussis toxin promoter. This variant allows bacteria to increase toxin production so that more severe clinical signs and symptoms manifest [3]. Unfortunately, we were able to analyze *B. pertussis* strains in only two infants because cultures were negative in 16 infants with PCR confirmed pertussis who were already receiving specific antibiotic therapy when specimens were collected, or because technical problems arose during sample collection.

Without seeking information from an in-depth molecular genetic analysis with standard protocols [4-6] we cannot say whether the pertussis strain isolated from our two patients differs immunologically from vaccine variants. One of our two patients was 1 month old and the other 5 months old. Neither infant had been vaccinated against pertussis nor had they received macrolides antibiotic treatment at the time we collected their nasopharyngeal aspirate specimens. Equally important, the isolates contained the variant *ptx-P3*, known as high-producing pertussis toxin [31], a variant that results in increased virulence and immune suppression. For this reason, we cannot exclude the possibility that both infants might have manifested the disease even if they had been vaccinated, because the vaccine-induced immunity would not have immunized them against the particular strain isolated.

The median age at onset in the 19 infants hospitalized for pertussis was low (around 70 days). This finding confirms international evidence showing that a vaccination strategy envisaging an additional dose at birth or maternal immunization is advisable to prevent disease in the first six months of life [20,28,32]. In accordance with published data we assume that improving surveillance and diagnosis would allow more accurate epidemiological assessments of pertussis and help to establish better vaccination strategies (times and age at vaccination).

Limitations of our study are the small number of infants enrolled, the fact that we could analyze *B. pertussis* strains in only two patients, and that nasopharyngeal washing is not the reference standard for detecting *B. pertussis* in culture.

## Conclusion

In conclusion, even during seasonal RSV outbreaks the differential diagnosis of bronchiolitis, especially in children younger than one year, should consider whooping cough. In the absence of exposure to ill contacts and local epidemiological surveillance, the major clinical factors for diagnosing whooping cough are typical paroxysmal cough, lymphocytosis, increased eosinophil numbers and absent abnormal chest sounds. Molecular analysis suggests that also in Italy *B. pertussis* strains differ from those included in the current vaccine. These findings might call for a change in the current pertussis vaccination, according also to the current strategies in the US and other countries that include maternal immunization with Tdap.

## Competing interests

The authors declare that they have no conflict of interest, other than any noted in the covering letter to the editor and non-financial competing interests.

## Authors’ contributions

AN Participated in the design of the study and wrote the paper. RN Participated in the design and coordination of the study and helped to draft the manuscript. PS Carried out the molecular genetic analysis and helped to draft the manuscript. AC Carried out the molecular genetic analysis. CS Carried out the acquisition of data. AP Carried out the virological analysis and helped to draft the manuscript. CS Carried out the virological analysis and helped to draft the manuscript, CM Helped to draft the manuscript, PP Helped to draft the manuscript, EB Performed the statistical analysis, MF Carried out the acquisition and participated to the statistical analysis. SP Carried out the acquisition and helped to draft the manuscript. FM Conceived of the study and participated in its design. Helped to draft the manuscript and revised the final version. All the authors read and approve the final manuscript.

## Pre-publication history

The pre-publication history for this paper can be accessed here:

http://www.biomedcentral.com/1471-2334/13/526/prepub
